# Value and Financial Toxicity of New Cancer Drugs

**Published:** 2016-05-01

**Authors:** Rita J. Wickham

**Affiliations:** Dr. Wickham is an adjunct faculty member at Rush University College of Nursing in Chicago, Illinois.

By now you’ve read Karen Herold’s excellent overview of the CLEOPATRA study in the January/February issue of JADPRO (2016). CLEOPATRA confirmed that PDT (pertuzumab [Perjeta], docetaxel, and trastuzumab [Herceptin]) was superior to docetaxel and trastuzumab (DT) to improve progression-free and overall survival for patients with HER2+ metastatic breast cancer (Baselga et al., 2012; Swain et al., 2015). However, Durkee and colleagues (2016) examined value and financial toxicity associated with PDT—issues not addressed in any CLEOPATRA publications.

During CLEOPATRA, investigators would not know the future price of pertuzumab, although trastuzumab cost approximately $70,000 a year. Durkee and others (2016) carried out Markov analyses to predict the "cost of progress." Averaged across treatment, the cost to insurers and patients receiving PDT would be *$4,649 per week*. For a median survival of 39.4 months for DT and 56.9 months for PDT, drug costs of $347,627 and $805,449, respectively, were predicted—plus other drugs, administration, laboratory, etc. If all 17,450 patients diagnosed with advanced HER2+ breast cancer each year got PDT or DT, now recommended by the National Comprehensive Cancer Network (NCCN, 2016) as first-line treatment for metastatic, HER2+ breast cancer, yearly costs would be billions, which Durkee concluded is not sustainable in the United States.

## VALUE AND COST/BENEFIT

Value and cost/benefit of cancer drugs became "front page" in a publication by 116 hematologists that discussed drug prices for chronic myelogenous leukemia (CML) and declared cancer drug prices are too high, untenable, may block patients’ access to therapy, and are detrimental to US health care (Experts in Chronic Myeloid Leukemia, 2013). High cancer drug costs began in 2001 with the approval of imatinib. Some drug companies argue that high research and development (R&D) costs must cover costs of unsuccessful drugs. However, most basic new drug research is done in universities and small biomedical firms. Then, pharmaceutical companies buy the new agent or biotech firm and pay clinical trials costs (Lexchin, 2012). Combined research efforts have increased 10-year CML survival from 20% to 80%, and many patients have near-normal life expectancies if they (and their insurer) continue to pay for their tyrosine kinase inhibitor. This is a pressing issue: Direct annual medical costs for cancer care are approximately $86 billion and are among the most rapidly growing spending disease groups (ASCO, 2015).

Imatinib initially cost ~$30,000 a year; R&D costs would have been recouped in 2 years (Love, 2013). It seems the current price of imatinib should be about the same or slightly lower (because of competition from new drugs), or perhaps a little higher to reflect modest inflation of the past decade. But imatinib costs > $150,000 per year, a > 500% price increase (GoodRx, 2016a)—2014 total sales were $4.746 billion dollars (PMLiVE, 2016).

Increasing drug costs are not limited to cancer; US branded prescription drug prices increased 10.9% in 2014 and 14.8% in 2015, while inflation rose 1.6% and 1.5%, respectively (Dennis, 2016; The World Bank, 2016). Drug spending was $297.7 billion in 2014; a third of that cost was new hepatitis C drugs. Cancer drugs cost significantly more in the Unites States than in other developed countries (CML Experts, 2013). Novartis had been able to protect patent rights and fend off generic versions of imatinib in the United States, but generics are available in other countries and one received US FDA approval in February 2016.

## SOLID TUMORS

The value dilemma is greater for drugs indicated for patients with advanced solid tumors, such as nivolumab (Opdivo), approved for patients with advanced nonsquamous non–small cell lung cancer (Borghaei et al., 2015). Patients in the pivotal phase III study, who ultimately died, were randomly assigned to receive *palliative* nivolumab or docetaxel. Response rates—evaluated after 2.5 months—were modest (as expected in previously treated patients with metastatic disease), 19% with nivolumab and 12% with docetaxel (p = .02). Overall rates of adverse effects were similar, but serious events with nivolumab included thyroid, pulmonary, hepatic, colon, and renal effects. Most patients progressed while on study and went on to receive ≥ 1 other therapies that probably influenced survival. This study was funded, developed, implemented, analyzed, and reported with pharmaceutical company sponsorship (Borghaei et al., 2015). Of the 792 patients enrolled in the study, 582 were randomized. The authors stated most of the other 210 patients "no longer met inclusion criteria" but provided no further explanation, and these patients were not included in the intent-to-treat analysis. Median overall survival with nivolumab was 12.2 vs. 9.4 months with docetaxel, a *statistically significant* difference (p = .002). Clinicians must help patients determine if this is *clinically* significant.

Nivolumab costs ~$15,000/month (GoodRx, 2016b). The cost to treat 100 patients for 3 months to identify 20 responders is ~$4,500,000, so the true cost of *palliative* nivolumab is $225,000 for each responder in the period. What is the value of 3 months’ survival in 20% of nivolumab-treated patients, who may experience major toxicity?

## AP ROLES

Advanced practitioners guide their patients through cancer diagnosis and treatment, and they educate and manage symptoms as well. But frank discussion of treatment costs is rapidly becoming integral to patient care. Advanced practitioners must familiarize themselves with ASCO’s Value Framework that emphasizes providing high-quality patient care and facilitates shared decision-making about the value of expensive diagnostic tests and new treatments, as well as becoming stakeholders to grapple with these issues (Schnipper et al., 2015).
